# Relationship between Household Air Pollution from Biomass Smoke Exposure, and Pulmonary Dysfunction, Oxidant-Antioxidant Imbalance and Systemic Inflammation in Rural Women and Children in Nigeria

**DOI:** 10.5539/gjhs.v5n4p28

**Published:** 2013-03-10

**Authors:** Oluwafemi Oluwole, Ganiyu O. Arinola, Godson R. Ana, Tess Wiskel, Dezheng Huo, Olufunmilayo I. Olopade, Christopher O. Olopade

**Affiliations:** 1 Center for Clinical Cancer Genetics, University of Chicago, Chicago, USA; 2 Center for Global Health, University of Chicago, Chicago, USA; 3 College of Medicine, University of Ibadan, Ibadan, Oyo State, Nigeria; 4 Pritzker School of Medicine, University of Chicago, Chicago, USA; 5 Department of Health Studies, University of Chicago, Chicago, USA; 6 Department of Medicine, University of Chicago, Chicago, USA

**Keywords:** biomass fuel, rural communities, oxidants, antioxidants, lung function, oxidative stress

## Abstract

**Background:**

Exposure to particulate matter from burning biomass fuels is believed to affect oxidant-antioxidant balance and to induce oxidative stress.

**Methods:**

Fifty-nine mother-child pairs from 59 households that used firewood exclusively for cooking in three rural communities in southwest Nigeria underwent blood test for albumin, pre-albumin, retinol-binding protein (RBP), superoxide dismutase (SOD), vitamins C, vitamin E, malondialdehyde (MDA) and C-reactive protein (CRP). Spirometry was performed and indoor levels of PM_2.5_ were determined.

**Results:**

Mean age (± SD; years) of mothers and children was 43.0±11.7 and 13.6±3.2, respectively. The median indoor PM_2.5_ level was 1575.1 µg/m^^3^^ (IQR 943.6–2847.0, *p*<0.001), which is substantially higher than the World Health Organization (WHO) standard of 25 µg/m^^3^^. The mean levels of pre-albumin (0.21±0.14 g/dL) and RBP (0.03±0.03 g/dL) in women were significantly lower than their respective normal ranges (1-3 g/dL and 0.2-0.6 g/dL, respectively, *p*<0.05). Similarly, the mean levels of pre-albumin (0.19±0.13 g/dL) and RBP (0.01±0.01 g/dL) in children were significantly lower than the respective normal ranges (1-3 g/dL and 0.2-0.6 g/dL, respectively, *p*<0.05). Mean serum concentrations of MDA in children (5.44±1.88 µmol/L) was positively correlated to serum concentrations of CRP (r=0.3, p=0.04) and negatively correlated to lung function (FEV_1_/FVC) in both mothers and children (both r=-0.3, *p*<0.05). Also, regression analysis indicates that CRP and SOD are associated with lung function impairment in mothers (-2.55±1.08, *p*<0.05) and children (-5.96±3.05, p=0.05) respectively.

**Conclusion:**

Exposure to HAP from biomass fuel is associated with pulmonary dysfunction, reduced antioxidant defense and inflammation of the airways. Further studies are needed to better define causal relationships and the mechanisms involved.

## 1. Introduction

Almost half of the world's population lives in rural areas where biomass fuels remain the main source of energy. It is now well established that burning of biomass fuels can cause household air pollution (HAP) and significantly elevate indoor exposure to particulate matter (PM) and high quantities of health-damaging pollutants, including carcinogens (Kocbach et al., 2009), that generate oxidants and free radicals (Ohyama, Otake, Adachi, Kobayashi, & Morinaga, 2007; Kampa & Castanas 2008). Chronic exposure to these compounds may elicit inflammation in the lungs, increase susceptibility to lung infection (Mott et al., 2005; Naeher et al., 2007), and induce cell membranes to undergo direct oxidant damage (lipid peroxidation), which can be measured with an assay of malondialdehyde (MDA). The adverse health effects of exposure to HAP may be explained by several mechanisms (Nel, Xia, Madler, & Li, 2006). One of the proposed mechanisms is oxidative stress resulting from one or a combination of oxidant/antioxidant imbalances, an excess of oxidants or a depletion of antioxidants (M. Cemek, Caksen, Bayiroglu, F. Cemek, & Dede, 2006), providing evidence for an etiologic role of oxidative stress in obstructive lung disease.

Women and children are particularly vulnerable to these adverse health effects because of the daily repetitive exposure to PM during cooking. Exposure to biomass smoke have been strongly linked to impaired pulmonary function (Saha, Rao, Kulkarni, Majumdar, & Saiyed, 2005; Regalado et al., 2006; Padhi & Padhy 2008; Fullerton et al., 2011), ranging from mild to moderate reductions in spirometric variables such as forced expiratory volume in one second (FEV_1_), peak expiratory flow (PEF), and forced expiratory flow (FEF_25-75_), which are measures of airways obstruction (Regalado et al., 2006; Torres-Duque, Maldonado, Perez-Padilla, Ezzati, & Viegi, 2008); as well as low antioxidants levels (Mishra & Retherford, 2007). Also a recent study linked increased exposure to outdoor air pollutants particularly PM_2.5_ (particles less than or equal to 2.5µm in aerodynamic diameter) with acute changes in biomarkers of inflammation such as CRP in healthy individuals (Rich et al., 2012). In addition, using MDA as marker of oxidant-antioxidant imbalance, women exposed to HAP from burning biomass fuels have significantly high levels of MDA (B. Isik, R. S. Isik, Akyildiz, & Topcu, 2005). The composition and quantity of antioxidants in the body represents an important determinant of individual susceptibility to HAP. Increased production of free radicals secondary to pollutant exposure may exceed the capacity of the antioxidant defense system, resulting in particulate-induced inflammation in the airways (Nel, 2005; Schlesinger, Kunzli, Hidy, Gotschi, & Jerrett, 2006), oxidative damage (Hiura, Kaszubowski, Li, & Nel, 1999; Mazzoli-Rocha, Fernandes, Einicker-Lamas, & Zin, 2010), and impaired lung function (Mazzoli-Rocha et al., 2008). HAP exposure has been reported to be one of the factors responsible for an estimated 2.5 million premature deaths and 3.7% of the loss of disability adjusted life years (DALY) every year in developing countries (Smith & Mehta, 2003; Emmelin & Wall, 2007).

In Nigeria, as in many other developing countries, most women and children who live in rural communities are poor and suspected to be nutritionally deficient. In addition, it has been reported that women in rural communities in Nigeria who cook regularly with biomass fuels had mean daily PM_10_ exposure of approximately 730 µg/m^3^; which is about 29-fold higher than the WHO daily limit of 25 µg/m^3^ (Ana, Adeniji, Ige, Oluwole, & Olopade, 2012). Since continuous exposure to high levels of HAP in the presence of poor nutrition may induce oxidative stress and trigger inflammatory responses (Mishra & Retherford, 2007; Montano et al., 2010), it is reasonable to assume that the women and children in rural communities in Nigeria who are nutritionally deficient and chronically exposed to HAP from biomass fuels are also at a considerable risk of oxidative stress. However, the level of HAP from biomass fuel use in rural households in Nigeria, its effects on pulmonary health, nutritional status and systemic inflammation is yet to be elucidated as little attention has been paid to this important public health issue. In view of this, we conducted a cross-sectional pilot study among women and children in households that cook with biomass fuels in rural communities in Nigeria. The objective was to investigate if a relationship exists between HAP from biomass smoke exposure and pulmonary health, oxidant-antioxidant imbalance and systemic inflammation. We also examined the relationship between oxidative stress (as measured by biomarkers of exposure) and lung function in this rural cohort.

## 2. Materials and Methods

### 2.1 Subjects

This cross-sectional study is part of an intervention pilot study to evaluate the extent, impact, and implication of HAP from biomass fuel use for cooking and monitored use of low-emission stoves on the health of women and children in southwest Nigeria. Fifty-nine mother-child pairs from 59 households that use biomass fuel almost exclusively for cooking in three rural communities (Ajibade, Eruwa, and Olorisaoko) near Ibadan, Nigeria, underwent detailed serum nutritional assessment and pulmonary function testing to investigate levels of serum oxidants/antioxidants following exposure to HAP. We performed spirometry in all mother-child pairs. To be eligible, subjects expressed willingness to participate in the study and were aged between 20–60 years (for mothers) and 7–17 years (for children). The lower age limit for children was set to ensure successful performance of spirometry, which requires subject cooperation.

The Institutional Review Boards at the Universities of Ibadan and Chicago gave ethical approval for the conduct of the study with approval numbers UI/EC/10/0045 and 10-263-B, respectively. All adult participants gave verbal and written consent for their participation and that of their children. Additionally, the children provided assent to participate.

### 2.2 Data Collection Methods

#### 2.2.1 Indoor Air Sampling during Cooking

Indoor air sampling was conducted in 59 households. Real-time measurement of PM_2.5_ was conducted in cooking areas before and during cooking using the pDR 1500 personal aerosol monitor and data logger (Thermo Scientific, Franklin, Massachusetts), which uses gravimetric and optically-based methods and compensates for many environmental variables during sampling. After calibration and equilibration, the instrument was set to cycle every minute for an hour during the cooking of evening meals. The sampler was positioned at the center of the kitchen, and it was placed approximately 0.5 meters from the ground and within a 1-meter radius of the plume arising from the pollution source to capture the exposure of the women and children during cooking.

#### 2.2.2 Pulmonary Function Tests

Pulmonary function tests were performed with the PC-based full function KoKo spirometer (nSpire Health, Inc. Longmont, Colorado) in accordance with the American Thoracic Society's (ATS) recommendations. Spirometry was performed at similar times of the day to minimize diurnal variation (Crapo, Morris, & Gardner, 1981); the spirometer was calibrated daily and operated within the ambient temperature. We measured forced vital capacity (FVC), forced expiratory volume in one second (FEV_1_), FEV_1_/FVC ratios, and peak expiratory flow rate (PEFR), which are reliable measures of airways obstruction.

#### 2.2.3 Blood Sample Preparation and Analytical Methods

Of the 59 mother-child pairs, 55 mother-child pairs consented to blood draw. Venous blood samples were collected in 10 ml Vacutainer EDTA tubes. All blood samples were processed at the Institute for Advanced Medical Research and Training laboratory at the University College Hospital, Ibadan, Nigeria. The serum samples were frozen and stored at -80°C until analysis was performed. Serum pre-albumin, albumin, transferrin, and retinal-binding protein (RBP) were determined using single immunodiffusion methods with results expressed as g/dL (Arinola & Ezeh, 2007).

Antioxidant enzyme superoxide dismutase (SOD) was assayed by the epinephrine autoxidation inhibition method (Magwere, Naik, & Hasler, 1997), while serum lipid peroxidation was assayed by the colorimetric reaction between MDA and thiobarbituric acid for measuring MDA (Kamal et al., 1989). Determination of vitamin C was performed using colorimetric method (Halliwell & Gutteridge 1995) while vitamin E determination was performed by high-performance liquid chromatography (Lang, Gohil, & Packer, 1986). Serum C-reactive protein (CRP) levels were determined by enzyme-linked immunosorbent assay (ELISA) immunoplate as described by the manufacturer (Immuno-Biological Labs. Inc.).

### 2.3 Data Analysis

Analyses were performed using STATA Version 12.0. The median (25th–75th percentile) value of household PM_2.5_ was determined. Mean values and standard deviations were calculated for lung function and serum nutritional biomarkers. Predicted normal values for lung function variables were obtained from the ATS recommendations. Student's *t*-test was used to compare lung functions and nutritional biomarkers with their corresponding normal values. In both women and children, bivariate Pearson's correlation coefficients (*r*) were used to determine the correlation between MDA and lung function, MDA and CRP, as well as SOD and CRP. To determine predictive factors of lung function impairment, multiple regression analysis was performed for %FEV_1_/FVC against variables of biomarkers of oxidative stress (MDA and CRP) and antioxidant variables (SOD, vitamin C and vitamin E). A *p*≤0.05 is considered statistically significant.

## 3. Results

### 3.1 Household Air Pollution and Lung Function

The sample included 118 subjects (59 mothers and 59 children). The mean age (±SD) of mothers and children were 43.0±11.7 and 13.6±3.2 years, respectively. Median concentrations of PM_2.5_ in the selected 59 households during cooking was 1575.1 µg/m^3^ [IQR 943.6–2847.0 µg/m^3^] and was significantly higher than WHO standards of 25 µg/m^3^ (*p*<0.001). Mean FEV_1_ was significantly lower than mean predicted FEV_1_ in both mothers (1.94±0.51 vs. 2.79±0.36 L/s, *p*<0.05) and children (1.76±0.54 vs. 2.57±0.66 L/s, *p*<0.05) (Table 1).

**Table 1:** Lung function parameters in exposed mothers and children

	Mothers (N=59)			Children (N=59)		
	
	Predicted Normal Value (mean±SD)	Best Effort (mean±SD)	*%* Predicted Normal Value^†^	Predicted Normal Value (mean±SD)	Best Effort (mean±SD)	*%* Predicted Normal Value^†^
FVC (L/s)	3.32±0.40	2.60±0.45	78.63±10.99	2.77±0.72	2.25±0.68	80.09±28.65
FEV_1_ (L/s)	2.79±0.36	1.94±0.51	69.78±16.58	2.57±0.66	1.76±0.54	69.29±15.01
FEV_1_/FVC (%)	0.84±0.29	0.74±0.13		0.93±0.24	0.81±0.12	
FEF_25-75_ (L/s)	3.08±0.71	1.87±0.81	59.32±25.21	3.11±1.03	1.95±0.99	64.10±30.28
PEFR (L/s)	6.11±0.31	3.52±1.45	58.47±23.21	5.78±1.17	3.25±1.48	57.97±24.59

† *p*-values <0.05 for all variables when compared with the predicted normal values

Abbreviation: FVC–Forced vital capacity; FEV_1_–Forced expiratory volume in one second; FEF–Forced expiratory flow; PEFR–Peak expiratory flow rate

### 3.2 Serum Biomarkers

Table 2 shows the summary of serum biomarkers of nutrition and antioxidants. Mean serum concentration levels of pre-albumin and RBP were below the lower limit of normal ranges, while concentration of albumin, transferrin, vitamin C, and vitamin E were all within the normal ranges in both mothers and children. Additionally, mean serum CRP concentrations in mothers (3.79±1.89 µg/ml) and children (3.56±1.54 µg/ml) were higher than normal range (1-3 µg/ml, *p*=0.39 and *p*<0.05, respectively). Mean serum MDA concentrations in mothers (5.90±2.40 µmol/L) and children (5.44±1.88 µmol/L) were also higher than normal range (<4.5 µmol/L, *p*<0.05 for both).

**Table 2:** Nutritional biomarkers in mothers and children

		Mothers				Children			
	
	Ref. range	N	Mean (±SD)	Min-Max	%Def.	N	Mean (±SD)	Min-Max	%Def.
Albumin (g/dL)	34–54	55	43.07±5.99	30–60	1.8	55	43.41±7.24	37–68	0
Transferrin (mg/dL)	1.2–2.6	55	1.74±0.53	0.92–2.8	5.5	54	1.61±0.56	0.7–2.8	11.1
Pre-albumin (g/dL)	1–3	55	0.21±0.14*	0.04–0.60	100	54	0.19±0.13*	0.04–0.60	100
RBP (g/dL)	0.2–0.64	55	0.03±0.03*	0.00–0.17	100	54	0.01±0.01*	0.00–0.05	100
SOD (Unit/L)	2.9–6.7	55	2.53±0.99	1–5.8	41.8	54	2.39±0.99	1–5.9	50.9
Vitamin C (mg/dL)	0.4–1.5	42	1.26±0.95	0.68–7.13	0	40	1.04±0.30	0.58–2.11	0
Vitamin E (mg/dL)	5–18	42	13.64±2.34	9.11–17.32	0	40	12.46±2.79	7.24–17.98	0

%Def. = % of subjects with values lower than the lower limit of normal range

* Significantly lower when compared to the lower limit of normal range

### 3.3 Relationship between Particulate Matter, Antioxidants, Oxidants, Lung Function, and Inflammation

**Figure 1:**
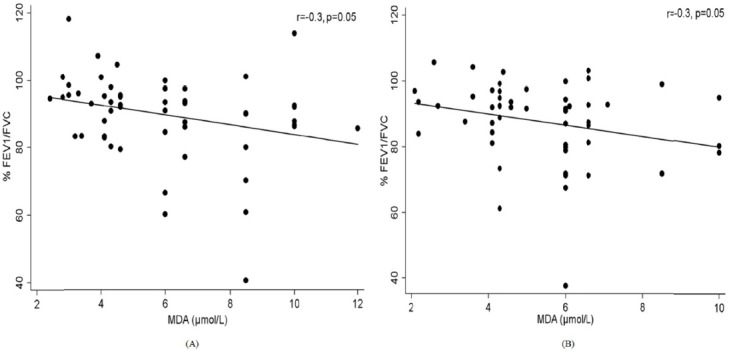
Association between lung function and lipid peroxidation (MDA) in mothers (A) and children (B)

**Figure 2:**
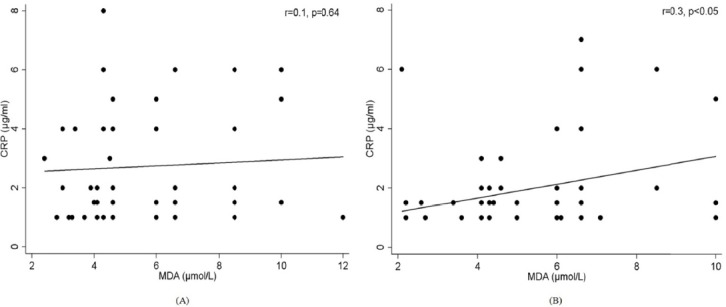
Association between MDA and marker of inflammation (CRP) in mothers (A) and children (B)

Correlation analysis showed no significant association between PM_2.5_ concentration levels and serum oxidant parameters. Also, no significant association was observed between PM_2.5_ concentration levels and FEV_1_ in mothers and children (*r*=0.08; *p*=0.57 and *r*=0.60; *p*=0.65, respectively). However, MDA concentration was negatively associated with FEV_1_/FVC in mothers and children (both *r*=-0.3, *p*=0.05; Figure 1). Assessing relationships between lipid peroxidation and propensity for inflammation, MDA showed a significant positive correlation with CRP only in children (*r*=0.3, *p*<0.05) but no significant association was observed in mothers (*r*=0.1, *p*=0.64; Figure 2). The predictor variables obtained for lung function impairment are presented in Table 3. Regression analysis indicates that serum CRP and SOD concentration levels were strong predictors of impaired lung function in mothers and children, respectively.

**Table 3:** Regression analysis of lung function (FEV_1_/FVC) against serum oxidative biomarkers and antioxidants

	Mothers		Children	
	
	Regression coefficient (±SE)	*p* value*	Regression coefficient (±SE)	*p* value*
MDA (µmol/L)	-0.65±0.91	0.48	0.39±1.39	0.78
CRP (µg/ml)	-2.55±1.08	0.02	0.07±1.38	0.44
SOD (Unit/L)	4.20±3.18	0.12	-5.96±3.05	0.05
Vit C (mg/dL)	0.69±2.10	0.74	-20.50±19.89	0.31
Vit E (mg/dL)	1.18±0.85	0.17	0.17±1.97	0.93

* *p*-values after adjusting for age, height and exposure to tobacco smoke

## 4. Discussion

Exposure to HAP from biomass smoke has been implicated as a significant risk factor for the development of inflammation and respiratory diseases (Ghaffari & Taghizadieh, 2010; Hu et al., 2010). Although the underlying mechanisms for exposure-related injury are still being investigated, exposure induced-oxidative damage has been alleged to play an important role (Schlesinger et al., 2006; Romieu, Castro-Giner, Kunzli, & Sunyer, 2008). In this study, the median PM_2.5_ concentration levels were 1575.1 µg/m^3^, more than 60 times higher than the WHO 24-hour acceptable levels of 25 µg/m^3^. Also, lung function, pre-albumin and RBP were significantly lower than their respective normal values among exposed mothers and children. Similar to previous studies which showed a relationship between biomass smoke exposure and adverse health outcomes in developing countries (Barnes, Mathee, Thomas, & Bruce, 2009; Fatmi, Rahman, Kazi, Kadir, & Sathiakumar, 2010), our findings suggest that exposure to HAP from biomass fuels may have significant effects on lung function and nutritional status. However, long term studies with a control arm of individuals who cook with cleaner fuel are needed to confirm this relationship.

Exposure to biomass fuels is recognized as an important cause of impaired pulmonary function (Saha et al., 2005; Regalado et al., 2006; Padhi & Padhy, 2008). In the present study, we observed impaired lung function in both non-smoking women and children (Table 1). Several studies have also reported reduction in lung function, characterized by FVC <80% of predicted value, in women chronically exposed to biomass smoke (Sumer, Turaclar, Onarlioglu, Ozdemir, & Zwahlen, 2004; Regalado et al., 2006; Revathi, Kutty, & Annamalai, 2012; Kurmi et al., 2013). Similar reductions in FVC and FEV_1_ have been observed in children living in homes that used biomass fuel for cooking (Rinnie et al., 2006). The impaired lung function that was observed in both mothers and children in this study may be due to PM_2.5_ that is present in biomass smoke, which is believed to induce oxidative injury to the lungs through their ability to form free radicals and cause airway inflammation (Romieu et al., 2008).

The study also demonstrates pro-oxidative potential effects of exposure to biomass smoke as indicated by elevated levels of MDA—a serum marker of systemic oxidative stress—and CRP in women and children. CRP is an indicator of generalized inflammation and most epidemiological studies have found that CRP level is significantly increased following exposure to cigarette smoke (Bazzano, He, Muntner, Vupputuri, & Whelton, 2003; Frohlich et al., 2003). Similarly, the positive association observed between MDA and CRP in our study suggests that continuous exposure to biomass smoke may also be inducing systemic oxidative stress in this rural population. This is consistent with other studies which found significantly increased levels of CRP following exposure to air pollution from particulate matter (PM) (Peters et al., 2001; Pope et al., 2004). This may be as a result of increased production of free radicals such as reactive oxygen species (ROS), which is exacerbated by the depletion of plasma antioxidants that protect against oxidative damage (Cemek et al., 2006; Pereira, Heck, Saldiva, & Rhoden, 2007).

The importance of antioxidant status in protecting against oxidative stress has been demonstrated in intervention studies linking supplementation of antioxidants to lower lipid peroxidation, especially among smokers (Block et al., 2002; Dietrich et al., 2002). Similar studies have also demonstrated that depletion of body antioxidant capacity either through low levels of non-enzymatic or enzymatic antioxidants rendered cells vulnerable to oxidative attack (Raijmakers, Peters, Steegers, & Poston, 2005; Forman, Maiorino, & Urseni 2010). Since the composition and quantity of antioxidants in the body represents an important determinant of individual responsiveness to toxic air pollutants (Romieu et al., 2008), we suggest that low levels of serum nutritional biomarkers and antioxidants in our study may have contributed to the systemic inflammatory responses and oxidative stress observed in our subjects. Serum pre-albumin and RBP concentrations were significantly lower than normal values. This is consistent with earlier reports suggesting that chronic exposure to biomass smoke may significantly lower antioxidant levels and contribute to nutrient deficiency and stunted growth in children (Mishra & Retherford 2007; Padhy & Padhi 2009).

Depletion of pre-albumin is an indicator of poor nutritional status and has been showed to be associated with COPD, especially in heavy cigarette smokers (Gocmen et al., 2010; Obase et al., 2011). We also observed low levels of pre-albumin, which was not independently associated with reduced lung function. However, since nutritional and antioxidant availability in the body have impacts on individual levels of oxidative stress and lung function (Larcombe et al., 2008), we posit that the low level of pre-albumin in combination with other nutrient deficiencies may have indirectly contributed to the increased levels of CRP. Our results also confirmed the reports of studies that showed oxidative stress to be negatively correlated with lung function and play a role in enhancing systemic inflammation (Ichinose, Sugiura, Yamagata, Koarai, & Shirato, 2000; Rahman & Adcock, 2006), as we also observed with CRP. Additionally, low serum RBP concentration is an indicator of vitamin A deficiency (Reifen, 2002; Ambalavanan, Ross, & Carlo, 2005). This deficiency, which may be exacerbated by inadequate nutritional intake has been shown to induce systemic inflammation and aggravates existing inflammatory conditions (Reifen, 2002). Also, low serum vitamin A is an independent risk factor for inflammation in children (Gamble et al., 2004). Nearly all mothers and children in our study showed evidence of inflammation, as indicated by elevated CRP levels. Although we did not assess vitamin A status in our subjects, the low levels of RBP, a carrier for vitamin A in the blood (Baeten et al., 2004), suggests significant vitamin A deficiency and may have contributed to the inflammation and oxidative stress.

Chronic exposure to biomass smoke generally causes cellular injury via oxidative stress; and to combat oxidative stress, the body has to develop a strong antioxidant defense system (Romieu et al., 2008). An important member of this defense system is the enzyme SOD. In the present study, SOD levels in both mothers and children were below the lower limit of normal range with 42% of mothers and 51% of children falling below this limit (Table 2). SOD scavenges free radicals and can prevent smoke-induced inflammatory responses (Hoshino et al., 1990; Foronjy et al., 2006). However, epidemiological studies examining the relationship between serum SOD levels and exposure to smoke have showed conflicting results. Two such studies observed significantly low SOD activity in population exposed to biomass smoke (Padhy & Padhi, 2009; Montano et al., 2010). In other studies, SOD levels were observed to be higher in cigarette smokers when compared to controls (Yokus, Mete, Mete, Cakir, & Toprak, 2005; Baharvand et al., 2010). Our result also showed generation of oxidative stress as SOD showed positive correlation with CRP in mothers and also appeared to be a strong predictor of lung function in children (Table 3). This indicates that SOD may have relevance as a potential biomarker of systemic inflammation. Also, given the evidence of systemic oxidative stress in individuals with chronic exposure to biomass smoke (Barreiro et al., 2005), we believe that the decrease in SOD levels in our subjects may be due to either increased oxidative stress from HAP and/or nutritional deficiency, which collectively limits antioxidant defense.

One of the major limitations of this study is the lack of information on the dietary intake among the population sampled. Many epidemiological studies have suggested that consumption of fruits and vegetables may play important roles in neutralizing free radicals and in protecting against oxidative damage. The low levels of nutritional biomarkers below the lower limit of normal are highly suggestive of poor nutrition and antioxidant deficiency. Another limitation is the lack of control households where cleaner fuels are used for cooking in the community where the study was performed. This made it impossible for us to be certain if the adverse health effects, oxidative stress and inflammation we observed is related to exposure to biomass smoke. It is also likely that this malnourished study population has elevated CRP and MDA due to nutritional deficiency or unrecognized lung infection and not because of exposure to biomass smoke. However, our data confirm previous reports concerning the relationship between HAP exposure and systemic oxidative stress. While exposure to biomass smoke may be a plausible explanation for the observed impaired pulmonary function (predominantly obstructive lung disease) and oxidative stress, we believe a more detailed, large-sample-size case-control study involving biomass users and clean fuel users (e.g. liquefied petroleum gas or electricity) would be necessary to accurately establish the causal relationships and to control for other confounding variables.

Despite these limitations, our results showed that exposure to HAP from biomass smoke in the setting of poor nutrition and antioxidant deficiency may have significant health implications. Exposed women and children had reduced lung function and inflammation of the airways. Results from our study also underscore the role of imbalance between oxidants and antioxidants mechanisms in the development of oxidative stress and impairment of lung function.

In conclusion, the observed associations between HAP exposure, oxidative stress and decrease in pulmonary function suggest that HAP from biomass fuel has detectable adverse effects in exposed mothers and children, which may be worsened by inadequate antioxidant capacity. Since biomass fuel remains the main source of domestic energy in many rural communities in Nigeria, health impairments observed among exposed mothers and children in this study may be representative of the health conditions of women and children in rural communities, who are similarly exposed to biomass smoke. Thus the magnitude of this public health problem calls for immediate attention to mitigate this problem. While the best intervention for mothers and children in rural communities is to avoid or minimize exposure to HAP from biomass fuels, the present data suggest that they may also benefit from improved nutrition. Additional studies with a control group that relate dietary intake to antioxidant levels are needed to better understand the role of antioxidant defense system in protection against the damaging effects of exposure to HAP.
